# Coordinated Eph-ephrin signaling guides migration and axon targeting in the avian auditory system

**DOI:** 10.1186/1749-8104-7-29

**Published:** 2012-08-21

**Authors:** Michelle R Allen-Sharpley, Karina S Cramer

**Affiliations:** 1 Department of Neurobiology and Behavior, University of California Irvine, Irvine, CA, 92697-4550, USA

**Keywords:** Development, Brainstem, Auditory system, Axon guidance, Migration, Chick, Eph receptor

## Abstract

**Background:**

In the avian sound localization circuit, nucleus magnocellularis (NM) projects bilaterally to nucleus laminaris (NL), with ipsilateral and contralateral NM axon branches directed to dorsal and ventral NL dendrites, respectively. We previously showed that the Eph receptor EphB2 is expressed in NL neuropil and NM axons during development. Here we tested whether EphB2 contributes to NM-NL circuit formation.

**Results:**

We found that misexpression of EphB2 in embryonic NM precursors significantly increased the number of axon targeting errors from NM to contralateral NL in a cell-autonomous manner when forward signaling was impaired. We also tested the effects of inhibiting forward signaling of different Eph receptor subclasses by injecting soluble unclustered Fc-fusion proteins at stages when NM axons are approaching their NL target. Again we found an increase in axon targeting errors compared to controls when forward signaling was impaired, an effect that was significantly increased when both Eph receptor subclasses were inhibited together. In addition to axon targeting errors, we also observed morphological abnormalities of the auditory nuclei when EphB2 forward signaling was increased by E2 transfection, and when Eph-ephrin forward signaling was inhibited by E6-E8 injection of Eph receptor fusion proteins.

**Conclusions:**

These data suggest that EphB signaling has distinct functions in axon guidance and morphogenesis. The results provide evidence that multiple Eph receptors work synergistically in the formation of precise auditory circuitry.

## Background

The sensory epithelium of the auditory system is unique in that it contains an orderly representation of stimulus frequency that lacks information about auditory space. Our ability to localize sound sources relies on interaural time differences (ITDs) and interaural level differences (ILDs) encoded in auditory brainstem circuitry. Circuitry that detects ITDs is well characterized in the avian auditory brainstem (1), in which nucleus magnocellularis (NM) receives tonotopically arranged auditory afferents from the VIIIth nerve [1,2]. Each NM cell projects bilaterally to nucleus laminaris (NL), which contains neurons that have bitufted dendrites [3] and act as coincidence detectors that respond maximally when simultaneous input is received on both dorsal and ventral dendrites [4]. While axosomatic contacts are made on both sides, ipsilateral NM axon branches contact dorsal NL dendrites, whereas contralateral NM axon branches contact ventral NL dendrites [5-7]. The contralateral branches of NM axons terminate in delay lines in NL [8-10], so that the location of NL neurons receiving coincident bilateral input is correlated with the azimuth of the sound source [8,11,12]. 

**Figure 1:**
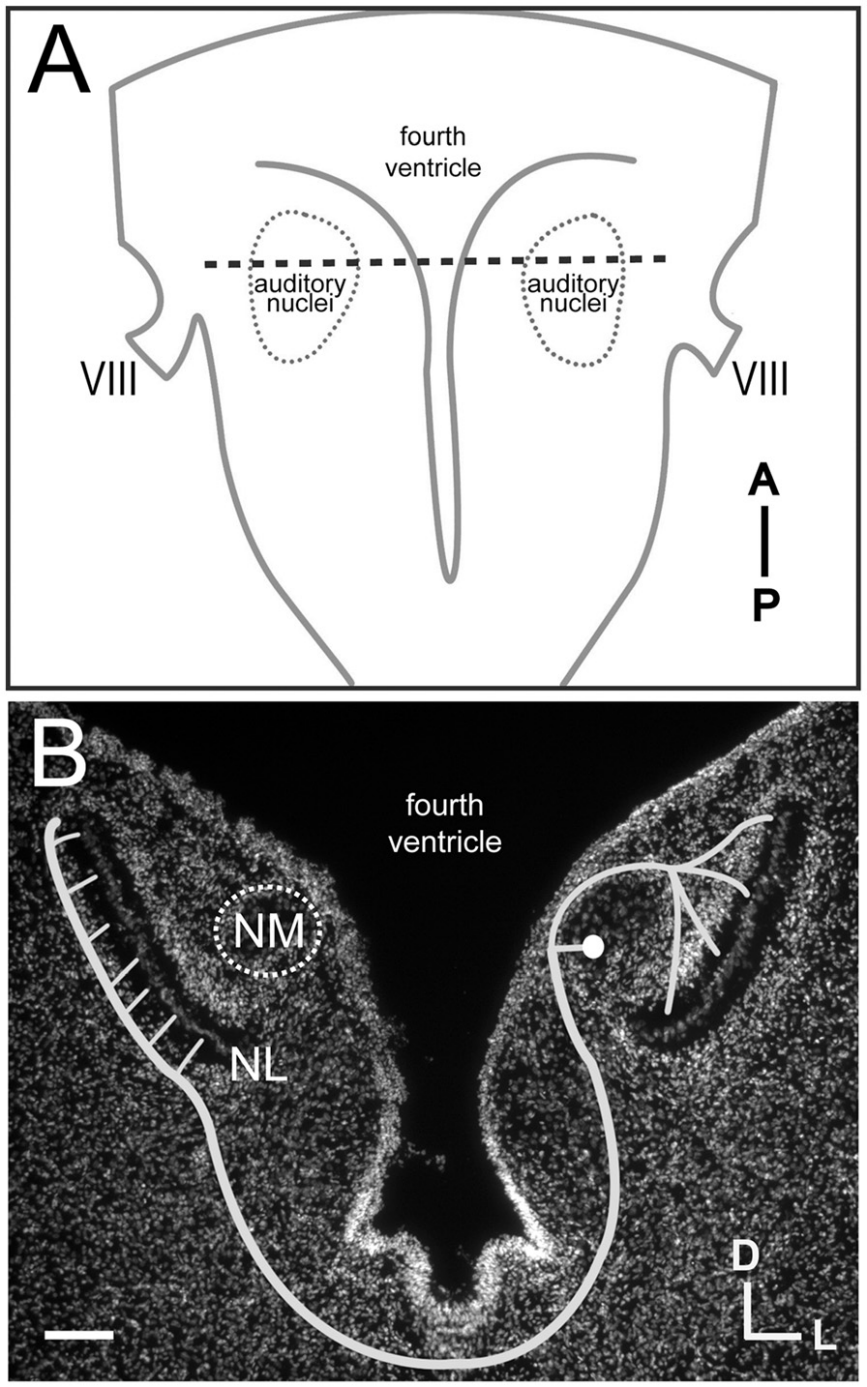
** Schematized NM-NL circuit.** (**A**) Illustration of a dissected chick embryo brainstem at E10 with auditory nuclei (including NM and NL) outlined bilaterally and the coronal plane of section for (**B**) indicated with a dashed line. (**B**) Coronal section of an E10 chick auditory brainstem with bisbenzimide nuclear staining. Bilateral projections from a single representative right-sided NM cell are schematized. Ipsilateral NM axon contacts dorsal NL neuropil with divergent branches of similar length, while contralateral NM axon contacts ventral NL neuropil with a delay line. Note the absence of cells in the neuropil surrounding NL cells where NM axons synapse. Scale bar = 0.5 mm in (**A**) and 100 μm in (**B**). VIII = eighth cranial (vestibulocochlear) nerve.

During development, contralaterally projecting NM axons cross the midline at E4 [13] and reach their target between E6 and E8 [14], at the same time that auditory brainstem nuclei begin to separate from the auditory anlage [14,15]. By E8, ipsilateral NM axon branches are visible. Synaptic connections from NM to NL form by E10, and axonal and synaptic refinement follow over the next several days [7,15,16]. Axons projecting from NM to NL are able to discriminate between the ventral versus dorsal dendrites of target cells with very few, if any, errors [11,17,18]. We have previously reported that Eph-ephrin signaling, known to be important for axon guidance and cell migration in other systems [19-25] has several distinct roles in the establishment of the NM-NL circuit [26-28].

Eph receptors comprise the largest class of receptor tyrosine kinases, and along with their ephrin ligands, are categorized into A and B subclasses based on structural similarity and binding affinities [29,30]. In general, EphA receptors bind ephrin-A ligands, and EphB receptors bind ephrin-B ligands, with two exceptions in which EphA4 also binds ephrin-B ligands [29,31] and EphB2 also binds ephrin-A5 [32]. Both receptor and ligand are membrane bound, with cell-cell interactions resulting in bidirectional signaling [33,34] and either attractive or repulsive cues to axons or migrating cells. The cell bearing the Eph receptor undergoes “forward” signaling, while the cell bearing the ephrin undergoes “reverse” signaling [35]. Embryos with impaired Eph-ephrin signaling exhibit a range of auditory defects including altered cochlear innervation and function [36,37], abnormal axon targeting in the auditory brainstem [17,38-41] and significantly impaired hearing [42,43].

At E10, when NM-NL contacts form, EphA4 is highly expressed in the dorsal, but not ventral, neuropil of NL [44]. In our previous study, we found that EphA4 misexpression resulted in a significant number of dorsoventral targeting errors from NM to contralateral NL [17]. However, the majority of NM axons terminated in their appropriate target region, suggesting that proper targeting relies on the coordinated functions of several molecules during development. We previously showed that several other Eph family proteins are expressed in NM and NL at the time that projections form [27]. Notably, EphB2 is expressed in both the dorsal and ventral NL neuropil, as well as in NM axons. In this study we tested whether EphB2 acts to segregate NM axons to appropriate dendritic regions of NL. We misexpressed EphB2 in NM cells using plasmid transfection or injected soluble Eph fusion proteins to inhibit forward signaling through Eph receptors in the region of NM axon growth, then examined the effects on the NM-NL pathway. We found that altering Eph-ephrin forward signaling during development of the NM-NL circuit resulted in impaired axon targeting, and that both EphA and EphB classes play a role in segregation of binaural inputs. Additionally, our studies suggest that EphB signaling is needed for the morphologic maturation of these auditory nuclei at several stages of embryonic development.

## Results

### Transfection is limited to auditory nuclei and extensive in NM cells

**Figure 2:**
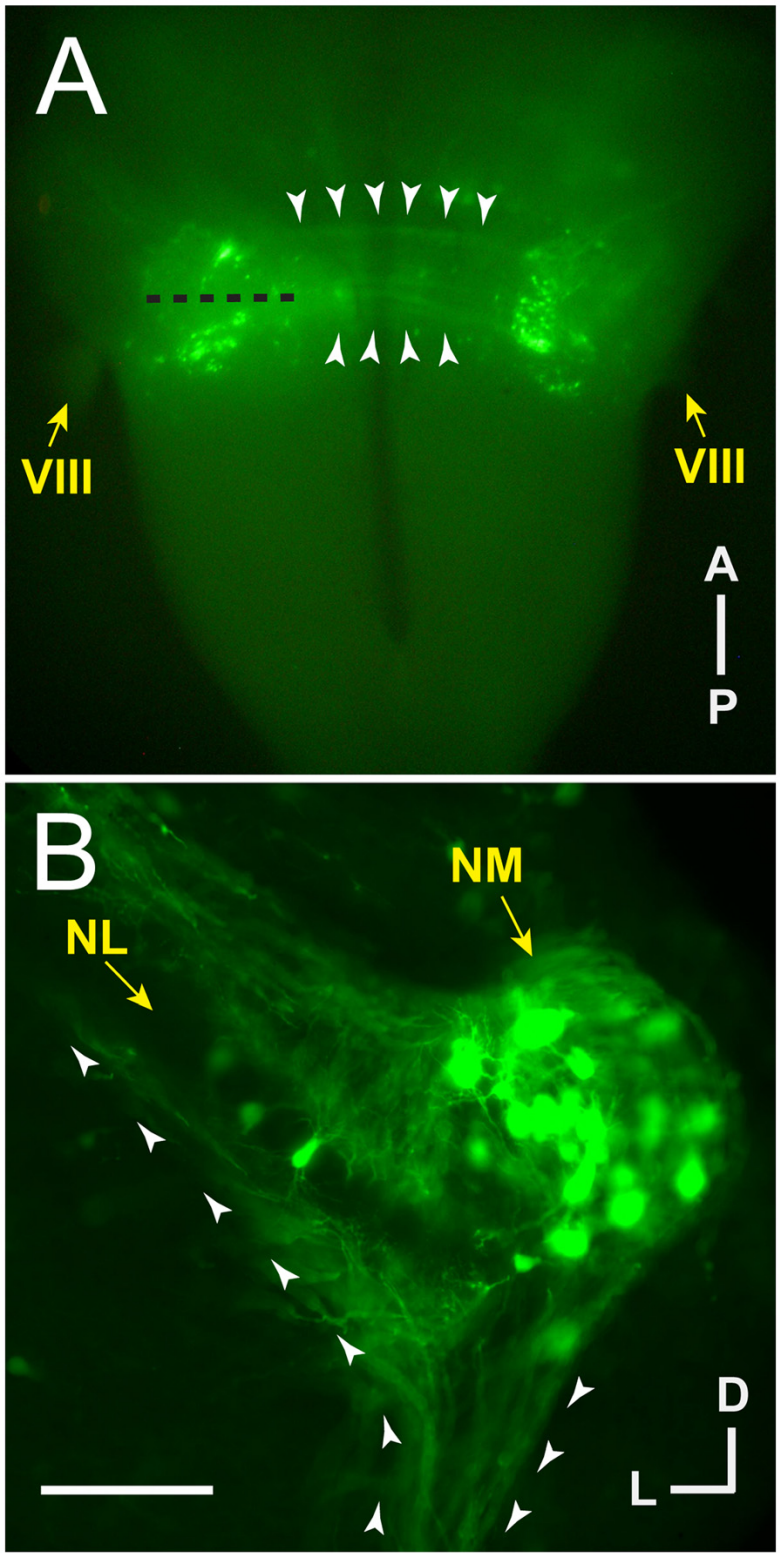
** Transfection is limited to auditory nuclei as seen by EGFP fluorescence.** (**A**) Dissected chick brainstem at E10 following *in ovo* electroporation at E2. Bilateral auditory nuclei are EGFP positive, as are the axons connecting NM to contralateral NL (white arrowheads). (**B**) Coronal section of E10 chick brainstem (left side) at approximately the level shown by a black dashed line in (A) shows transfection is extensive in but not limited to NM cells and their axons. In the section shown here, three NL cells and their bipolar tufts of dendrites within the cellular monolayer are visible by EGFP fluorescence. (A, B) White arrowheads delineate the margins of contralateral NM axons, and are oriented in (B) to indicate anterograde direction from NM origin to contralateral target. Scale bar = 1 mm in (A) and 100 μm in (B).

As we have previously shown [17,45], transfection of auditory cell precursors by *in ovo* electroporation at E2 leads to prolonged plasmid expression as shown by EGFP reporter expression and by immunolabeling of protein encoded by the transfected plasmid. Transfection was directed focally to the auditory brainstem precursors at E2 by placing the electrodes at the level of r5 [46]. At E2, NM and NL cells are undergoing their final mitotic divisions and rhombomere boundaries are visible. Following electroporation at E2, inspection of embryos revealed normal rhombomere morphology at E3. EGFP was visible and limited to the auditory region of whole brainstems dissected at E10 (Figure 2A). We found extensive transfection throughout NM that included cell bodies and axons, with more limited transfection seen in NL. In cases with NL cell transfection (Figure 2B), the number of transfected NL cells were outnumbered over 10:1 by NM transfected cells. A total of 24 electroporated embryos met inclusion criteria (described in Methods) and were used in the axon targeting analysis, while 57 were used at least in part for anatomical analyses. 

### Misexpression of EphB2 impairs axon targeting and NL morphogenesis

**Figure 3:**
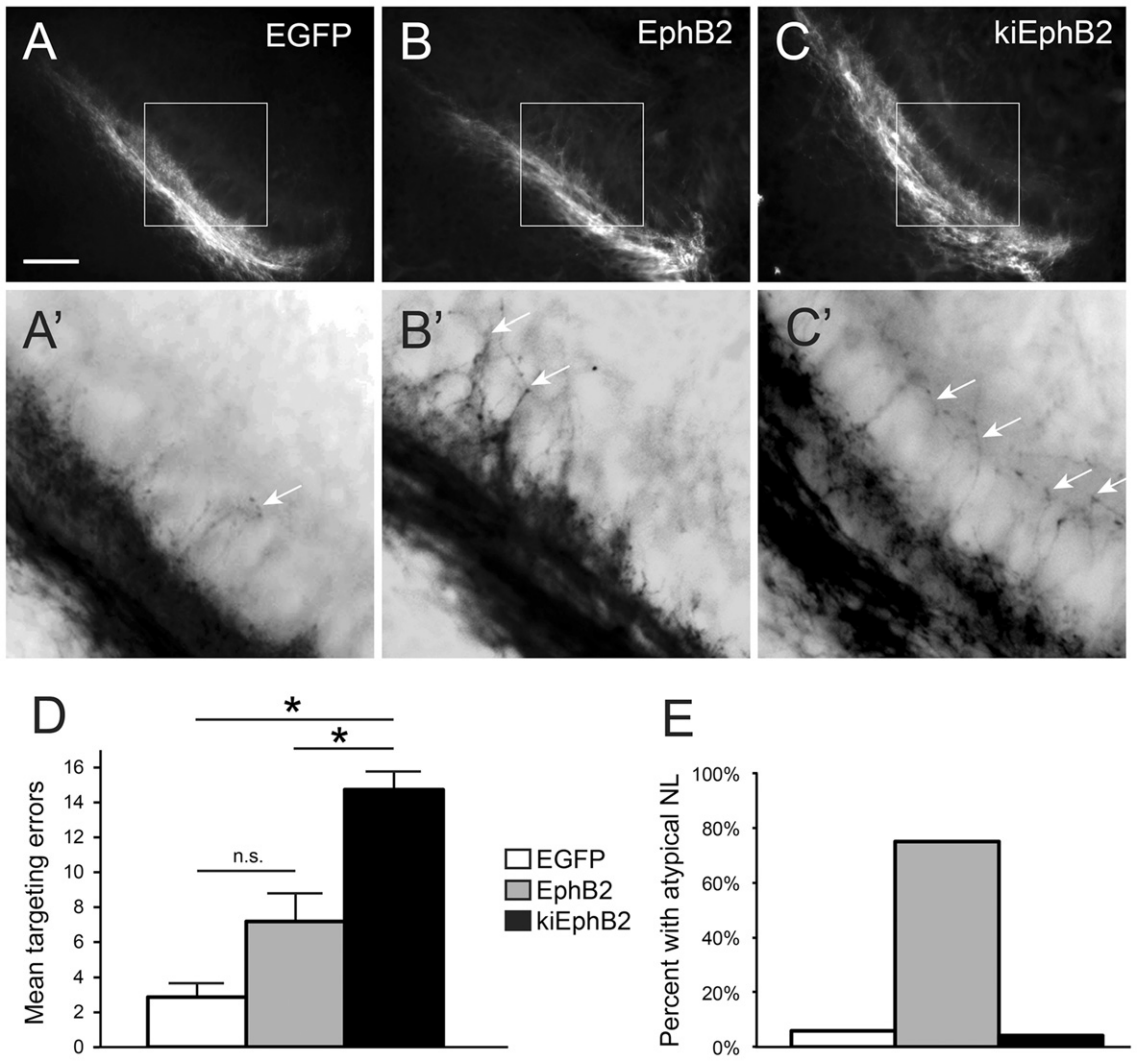
** Targeting errors are increased when EphB2 is misexpressed during development.** (**A**-**C**, **A’**-**C’**) RDA trace of left-sided NL in E10 embryos following E2 transfection of EGFP control (**A**, **A**’), full-length EphB2 (**B**, **B**’), or kiEphB2 (**C**, **C**’) plasmid. (**A**, **B**, **C**) are original 20X images with the outlined box expanded and color-inverted in (**A**’, **B**’, **C**’) to highlight fine processes of single axons crossing the NL cell body layer inappropriately (white arrows). (**D**) Quantification of the mean number of axon targeting errors per group with SEM bars. (**E**) Percentage of embryos in each group demonstrating atypical features of NL morphology, including a reduced length and/or a disorganized cellular layer. Scale bar = 100 μm in (**A**-**C**) and 35 μm in (**A**’-**C**’).

Axon targeting in the NM-NL pathway was analyzed in E10 embryos after transfection. Each embryo was considered a single data point, and targeting errors were quantified across the central region of each right and left NL. As expected, control embryos with EGFP transfection showed few contralateral NM axons in the dorsal region of the NL neuropil (Figure 3A, A’), with 2.85 ± 0.80 errors per 400 μm of NL (n = 7; Figure 3D). In embryos transfected with a full-length wild type EphB2 (Figure 3B, B’) the mean number of targeting errors was 7.17 ± 1.70 per 400 μm of NL (n = 6; Figure 3D), which did not significantly differ from EGFP controls (*P* = 0.09; Student’s *t*-test). To test the effect of reduced EphB2 signaling, we examined embryos transfected with kiEphB2, which acts as a dominant negative to inhibit forward signaling by interfering with endogenous receptor function [47] but acts similar to EphB2 in its potential to activate reverse signaling. We found that misexpression with kiEphB2 resulted in a significant increase in axon targeting errors from NM to contralateral NL (Figure 3C, C’). The mean number of targeting errors was 14.73 ± 1.63 per 400 μm of NL (n = 11; Figure 3D), which was significantly greater than that seen in control embryos (*P* < 0.0001) or in EphB2-transfected embryos (*P* = 0.0028). The significant increase in errors associated with kiEphB2 compared to EphB2 suggests that EphB2 forward signaling selectively affects targeting of NM axons to appropriate dendritic targets in NL and that it is a cell-autonomous effect since NM (axons) rather than NL (target) cells were transfected. 

We examined the morphology of auditory brainstem nuclei in electroporated embryos. During data collection, abnormal NL morphologies were observed in which NL was either not organized into a flattened cellular monolayer and/or was reduced in length. In some instances, ipsilateral NM and NL were positioned in closer proximity to each other than expected for the age examined. The percentages of embryos within each group meeting any of these criteria for atypical NM-NL morphology are shown in Figure 3E. Overall, 75% (12 of 16) of embryos expressing functional EphB2 plasmids had an atypical NL morphology compared to 6% (n = 17) and 4% (n = 24) of embryos transfected with EGFP alone or kiEphB2, respectively. Unlike EphB2 misexpression studies, there were no abnormal morphologies associated with kiEphB2 misexpression, suggesting that these effects arise from an increase in EphB2 forward signaling. In EphB2 transfected embryos, though the average increase in errors was not significant compared to controls, those with the greater number of targeting errors were also those with appreciable but not exclusionary abnormalities in NL morphology.

### Eph fusion proteins as inhibitors of Eph signaling

**Figure 4:**
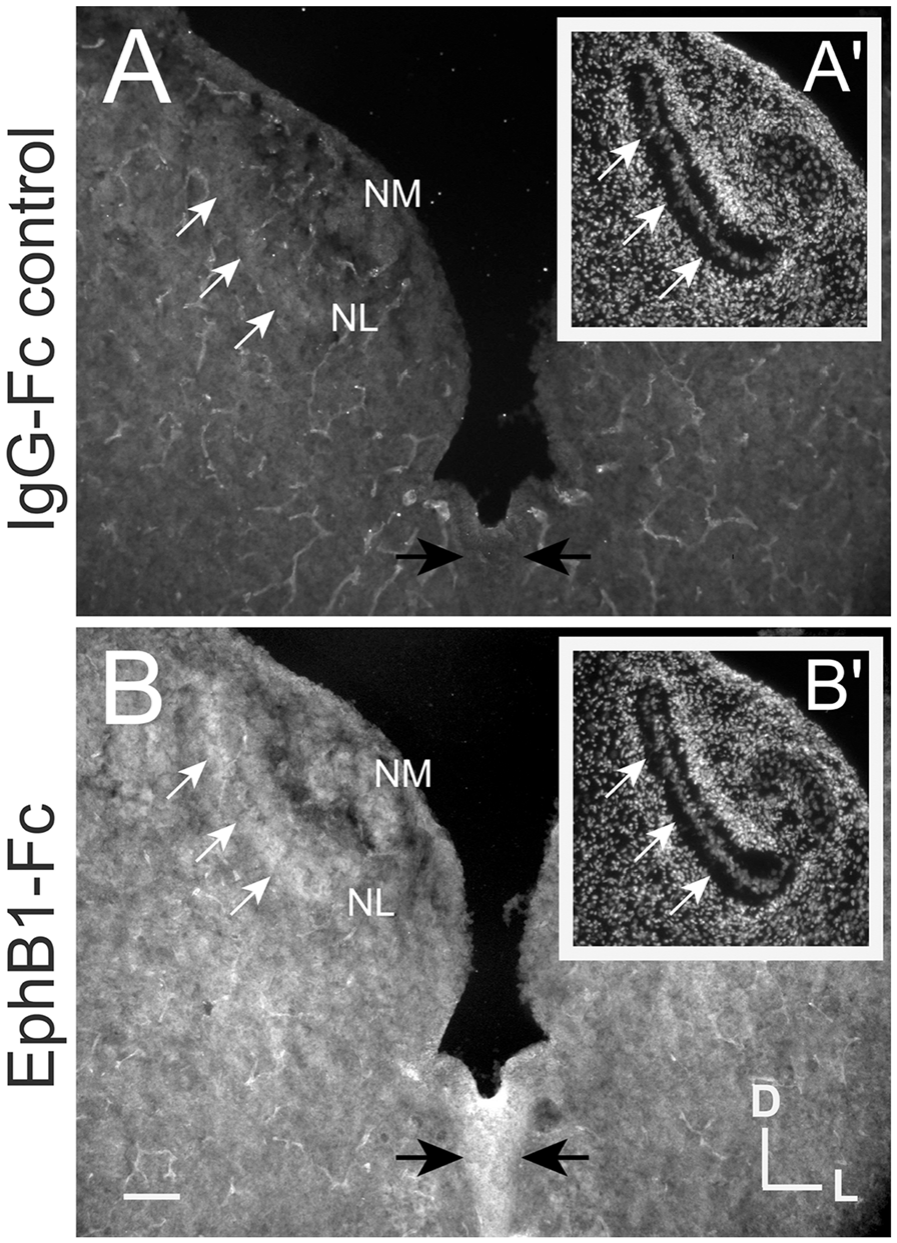
** Eph receptor fusion protein binds endogenous ephrin ligands.** (**A**) and (**B**) are adjacent coronal sections of an E10 embryo, incubated with either IgG-Fc (**A**, **A**’) or EphB1-Fc (**B**, **B**’) fusion proteins. Bisbenzamide nuclear staining of the same section (**A**’ and **B**’ for **A** and **B**, respectively) is used to identify NM and NL. White arrows indicate the ventral border of NL neuropil, and black arrows indicate the margins of the midline, both are locations where ephrin-B ligands are highly expressed at this age. EphB1- Fc binds NL and midline regions (**B**), whereas IgG-Fc does not (**A**). Scale bar = 100 μm.

The electroporation studies suggest that EphB2 forward signaling regulates both axon guidance and auditory brainstem morphogenesis. We further tested this possibility by inhibiting forward signaling during the time that NM axons approach their contralateral NL target. We used unclustered soluble Eph receptor fusion proteins to inhibit forward signaling by endogenous ligands. The fusion proteins only differ at their C-terminal region, either having or lacking an Eph receptor extracellular domain, with an identical IgG-Fc region at the N-terminus. We first demonstrated that EphB1-Fc has the expected binding properties based on known expression patterns of ephrin-B ligands in the chick auditory brainstem (Figure 4). Adjacent sections of cryosectioned tissue were incubated with either EphB1-Fc or IgG-Fc, followed by labeling with the same secondary fluorescent anti-Fc antibody. Nuclear counterstaining with bisbenzamide was used to identify NL (Figure 4A’, B’). While IgG-Fc treatment showed no immunolabeling (Figure 4A), we found that EphB1-Fc preferentially binds regions of the auditory brainstem where ephrin-B ligands are highly expressed at the age examined, including the midline and NM-NL neuropil (Figure 4B).

### Broad inhibition of Eph signaling impairs axon targeting and NL morphogenesis

**Figure 5:**
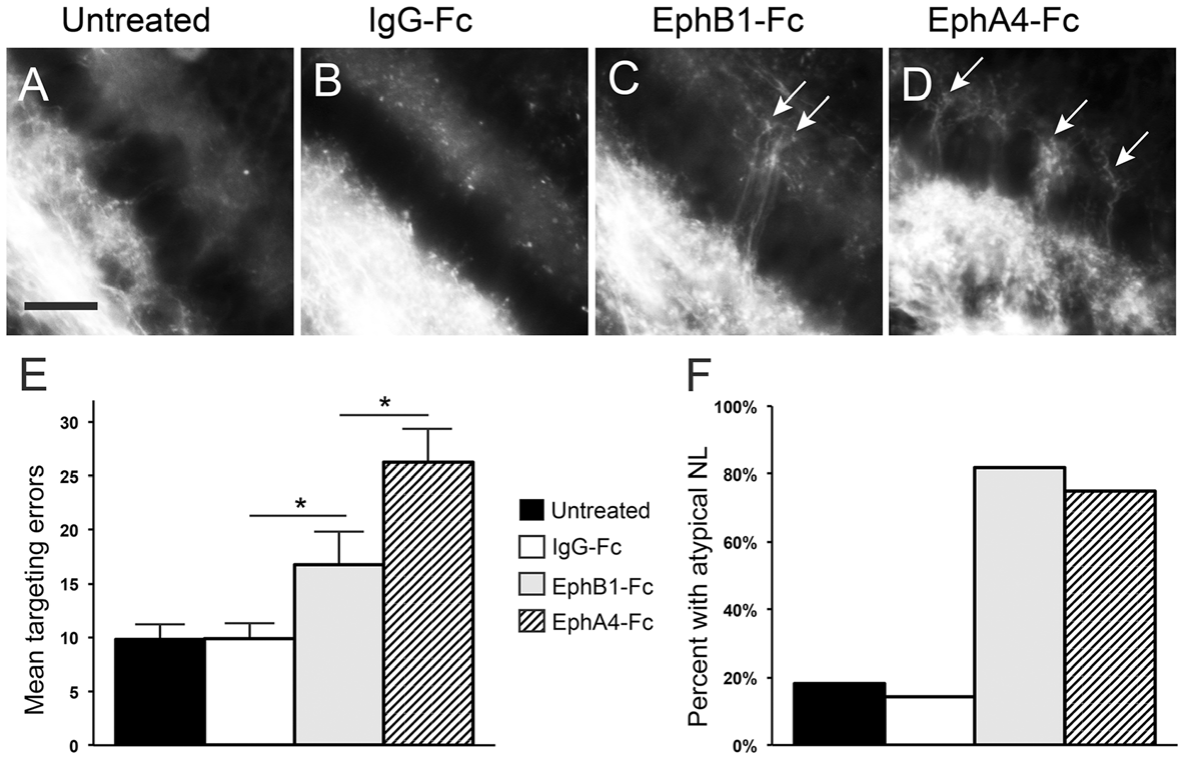
** Treatment with Eph receptor fusion protein results in increased targeting errors and atypical NL morphology.** (**A**-**D**) RDA tracings of contralateral-projecting NM axons at E10 in untreated embryos (**A**) or embryos treated during E6 to E9 with IgG-Fc control (**B**), EphB1-Fc (**C**), or EphA4-Fc (**D**). Axonal processes inappropriately cross the NL cell body layer, terminating in the dorsal NL neuropil in embryos treated with EphB1-Fc or EphA4-Fc (white arrows, **C**, **D**). (**E**) Quantification of mean axon targeting errors and (**F**) percentage of embryos with an atypical NL (including either atypical NL morphology or atypical NL border or both). Scale bar = 25 μm.

Because soluble fusion proteins are subject to degradation, the competitive inhibition assays were all performed in an *ex ovo* preparation that provided access to the brainstem at later embryonic ages and permitted injections in a localized region over the course of several days. Fusion proteins solubilized in PBS were injected into the developing hindbrain and fourth ventricle for four consecutive days from E6 to E9, when contralateral-projecting NM axons have already crossed the midline and are approaching their NL target [13]. Since it is possible that multiple Eph-ephrin pathways coordinate axon targeting of this pathway, we performed differential inhibition of the subclasses. EphB1-Fc was used to exclusively inhibit EphB forward signaling because EphB1 only binds ephrin-B ligands. EphA4-Fc was used to inhibit all EphA and EphB forward signaling because, in addition to ephrin-A ligands, EphA4 binds ephrin-B2, a ligand for both subclasses of Eph receptors. Our goal here was to discriminate between the effects of the receptor subclasses, particularly whether EphB signaling was unique or overlapping with EphA signaling. For negative controls, we had an untreated group and a group that received injections of human IgG-Fc. A total of 29 samples were used in the axon targeting analysis and 35 were used in the analysis of anatomical measurements. Representative images and quantification of targeting errors for each condition are shown in Figure 5. 

Embryos with no injections that were prepared *ex ovo* had a mean of 9.80 errors ± 1.48 per 400 μm NL (n = 10; Figure 5A), an error rate that did not differ significantly from control embryos treated with IgG-Fc (Figure 5B; 9.86 ± 1.55 per 400 μm NL, n = 7; *P* = 0.99). In contrast, we found there was a significant increase in axon targeting errors from NM to contralateral NL after treatment with EphB1-Fc (Figure 5C; 16.75 ± 3.22 per 400 μm NL, n = 8, *P* = 0.04) and with EphA4-Fc (Figure 5D; 26.25 ± 3.09 per 400 μm NL, n = 4, *P* = 0.0009) as compared to IgG-Fc controls. The effect of EphA4-Fc was significantly greater than that of EphB1-Fc (*P* = 0.02; data summarized in Figure 5E). Often multiple axons were seen crossing together in EphA4-Fc treated embryos (Figure 5D) so the number of errors is likely underreported due to our conservative analysis. These data suggest that forward signaling through Eph receptors is necessary for appropriate axon targeting, and that the EphA and EphB receptor classes make individual contributions to axon targeting in the NM-NL pathway.

**Figure 6:**
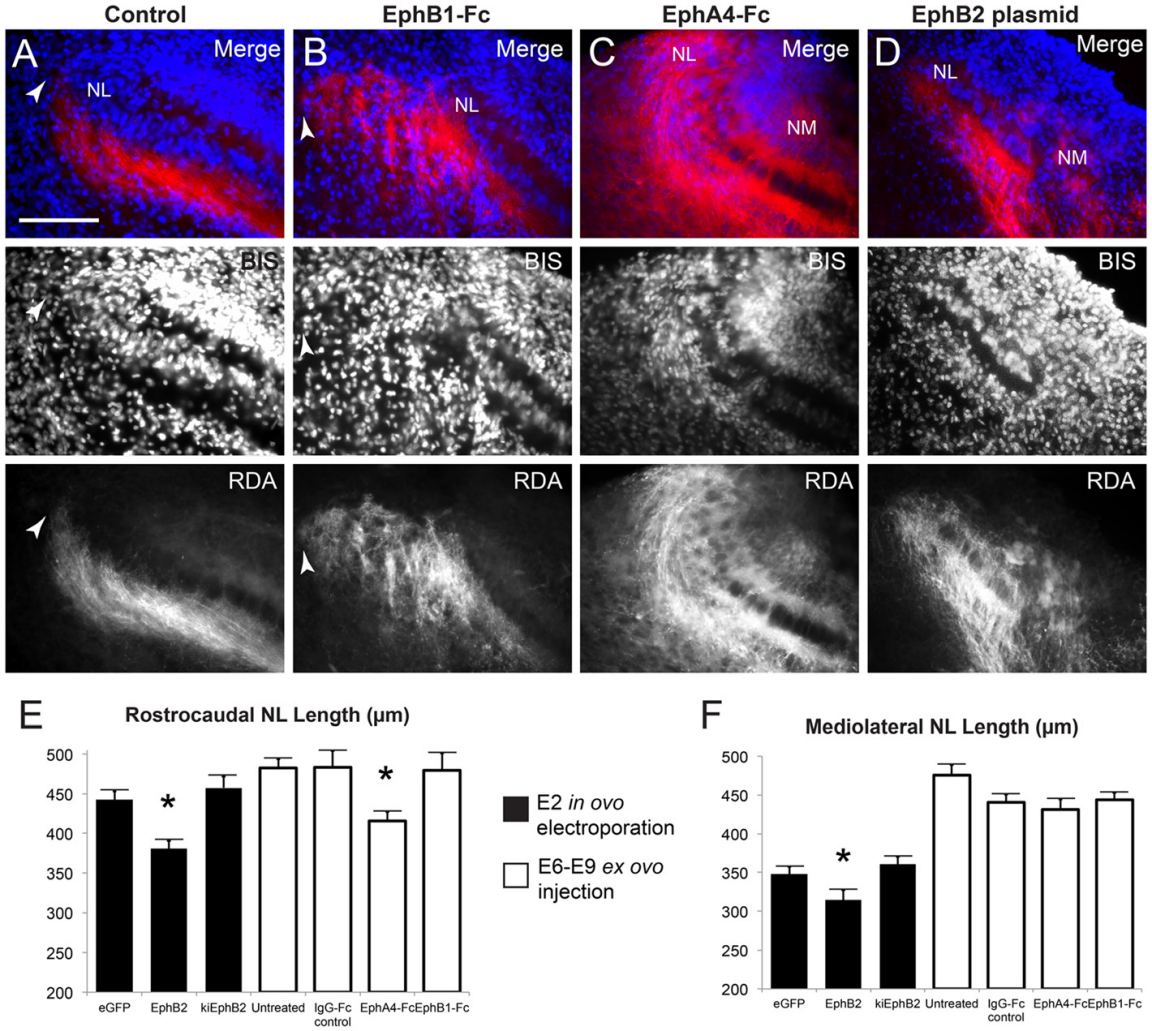
** Development of NL is affected by impaired Eph receptor signaling.** Representative 40X images of lateral NL following RDA midline tracing of E10 control embryos (**A**), embryos treated with EphB1-Fc (**B**), EphA4-Fc (**C**), or transfected with full-length EphB2 (**D**). Top panels are a merge of bisbenzamide (BIS, blue) nuclear counterstaining and RDA (red) fluorescence from labeled contralateral NM axons; both channels are shown individually in respective middle and lower panels. NL is normally an elongated, flattened layer with a lateral border (leftmost on these images) well defined by contralateral NM axons (white arrowhead in **A**). Treatment with EphB1-Fc (**B**) results in disorganized NM axons projecting lateral to the NL border (white arrowhead in **B**). Treatment with EphA4-Fc results in an abnormal NL morphology, where the mediolateral length is reduced and the lateral aspect is disorganized and not flattened (**C**). NM is normally found dorsomedial to NL and not within the same imaging field at 40X magnification, but the positioning of NM relative to NL is altered following transfection with full-length EphB2 at E2 (**D**) or treatment with EphA4-Fc from E6 to E9 (**C**). Measurements of NL (**E**, **F**) demonstrate the reduced extent of NL along both rostrocaudal (**E**) and mediolateral (**F**) axes when EphB2 is overexpressed from E2, and along the rostrocaudal axis (**E**) when all Eph receptors are inhibited with EphA4-Fc injection from E6 to E9. Scale bar = 100 μm, left is lateral and dorsal is up.

We found that, similar to plasmid electroporated embryos, NL was often disorganized following EphB1-Fc or EphA4-Fc injection. However, unlike the embryos subjected to plasmid electroporation, there were also appreciable targeting abnormalities of NM axons at the ventrolateral neuropil of NL that were not related to crossing the NL cell body layer. The percentages of embryos having any of these atypical morphologies are shown in Figure 5F. Overall, 82% (9 of 11) of EphB1-Fc and 75% (6 of 8) of EphA4-Fc injected embryos were atypical, compared to 17% of combined controls (n = 18). Of the EphB1-Fc treated embryos, a majority of the atypical morphologies (7 of 9) were limited to aberrant projections along the ventrolateral NL neuropil (Figure 6B) with a normal NL monolayer, whereas NL formation itself appeared affected more often in EphA4-Fc treated embryos (4 of 6; Figure 6C).

### Impaired development of auditory brainstem nuclei

When embryos were transfected with functional, full-length EphB2, the development of auditory nuclei was often impaired, with 75% (12 of 16) of transfected embryos showing abnormal NL morphology (Figures 3E, 6D). This observation contrasts greatly with controls, as only 6% (n = 17) and 4% (n = 24) of embryos transfected with EGFP alone or kiEphB2, respectively, showed abnormal morphology. There was wide variation in the observed range of morphological defects, from merely a reduced NL extent without disorganization (two cases) to a severe malformation of both auditory nuclei (two cases) where NM and NL could not definitively be identified. In total, six samples were excluded from axon targeting analyses for not meeting the necessary criteria, as NL was either not identifiable or not presenting as a monolayer with discrete ventral and dorsal neuropil. Similar changes in NL were seen with *ex ovo* inhibition of EphA and EphB receptors together, where 75% of the EphA4-Fc injection cases (n = 8) had some level of disorganization at the NL layer (Figures 5F and 6E), two of which were severe enough to exclude them from analysis of targeting errors. The remaining samples with acceptable axon tracing often displayed an unusual pattern of axonal projections that appeared to extend past NL and terminate in a laterally adjacent brainstem region. Of embryos receiving EphB1-Fc injections to inhibit EphB receptors alone, only 18% displayed an abnormal NL morphology, but aberrant laterally projecting NM axons were observed in 72% of the cases (n = 11; Figure 6B) and these axons were more numerous and appeared to extend farther than in cases with EphA4-Fc injection.

We found that 17% of the *ex ovo* control cases (n = 18) also displayed some aberrant lateral projections but to a much lesser extent than treated embryos. These aberrant lateral projections were never observed with any of the *in ovo* preparations. Thus, it is possible the impaired lateral targeting is permitted within the *ex ovo* preparation and is exacerbated by the inhibition of Eph signaling. Several anatomical variables were quantified, including rostrocaudal and mediolateral lengths of NL (Figure 6E, F) as well as distance and angle between NM and NL (not significant, data not shown). For embryos overexpressing wild-type EphB2, measures were consistently reduced across variables as compared to controls. The rostrocaudal and mediolateral extents of NL were both significantly reduced (p = 0.03 and *P* = 0.001 respectively). For embryos treated with EphA4-Fc, the rostrocaudal extent of NL was significantly reduced compared to IgG-Fc controls (*P* = 0.0169). An example of a severely reduced NL extent without NL disorganization is shown in Figure 6D, and a disorganized NL also having a reduced NL extent is shown in Figure 6C. Group data for rostrocaudal and mediolateral extents of NL are presented in Figure 6E, F. Internal controls were used to analyze *in ovo* versus *ex ovo* experiments separately to exclude any differences associated with the preparations.

## Discussion

In this study, we tested the role of EphB signaling in the guidance of NM axons to dorsal versus ventral dendritic regions of NL and in the morphogenesis of these auditory nuclei. We found that reduction of EphB2 forward signaling, either by kiEphB2 transfection or by local inhibition with soluble fusion proteins, resulted in a significant increase in the number of mistargeted contralateral NM axons as compared to controls. In addition, there were significantly more targeting errors when forward signaling through both EphA and EphB receptor subclasses were inhibited compared to inhibition of EphB receptors alone. While EphB2 electroporation, performed at E2, did not alter axon targeting, we found that it resulted in abnormal NL morphology in which NL was significantly shorter along both rostrocaudal and mediolateral brain axes than in controls. Morphological defects were also seen with fusion protein treatment, performed from E6 to E9. EphB1-Fc injections resulted in markedly aberrant NM projections at E10 that exceeded the contralateral NL boundary and projected laterally. EphA4-Fc injections resulted in malformed NL lamina, and aberrant laterally projecting axons, though less prominent than in EphB1-Fc treated embryos. NL was significantly shorter along the rostrocaudal axis with no change in mediolateral length compared to IgG-Fc injected and untreated controls. Together, these studies demonstrate several distinct functions for Eph proteins in the formation and connections of NM and NL.

### Forward EphB2 signaling guides NM axons

We previously showed that misexpression of EphA4 resulted in a significant number of targeting errors in which contralateral NM axons extended into the dorsal NL neuropil [17]. The expression of EphA4 in NL and not NM, together with the observation that EphA4 and kiEphA4 had similar effects, suggested a non-cell-autonomous role in which EphA4 in the target stimulates NM attraction through reverse signaling in NM axons. While those studies were initially motivated by the asymmetric expression of EphA4 in NL neuropil regions [44], the finding that many NM axons retained their appropriate targeting after misexpression prompted us to consider the potential roles of other Eph proteins. While EphA4 was unique among these proteins in its asymmetry, both EphB2 and ephrin-B1 showed high expression levels in dorsal and ventral NL neuropil, and EphB2 and ephrin-B2 were seen in NM axons [27].

The electroporation studies carried out here examined the role of EphB2 in NM axons. We found that kiEphB2, but not EphB2, increased targeting errors by NM axon branches into contralateral dorsal NL. Because kiEphB2 decreases forward signaling while EphB2 increases it, our results suggest that forward signaling through EphB2 in NM axons is needed for the restriction of NM axons. The observation that EphB2 overexpression in NM axons did not have an opposite effect likely reflects the normally high precision in NM axon segregation. Together with our EphA4 study, these results suggest that bidirectional Eph signaling regulates the binaural segregation of NM axons in NL.

Based on our previous expression analysis [27], forward signaling through EphB2 in NM axons could be elicited through (1) ephrin-B1, which might provide chemorepulsive signals to NM axons entering the ventral NL neuropil, and/or (2) by ephrin-B2, which is expressed in NL cell bodies and could act as a barrier to axons to prevent them from reaching the dorsal neuropil layer. This latter possibility could provide an effective barrier for both ipsilateral and contralateral NM-NL projections. As ephrin-B2 is expressed in NM axons [27], an attractive reverse signaling cue from EphA4 in the dorsal NL neuropil would then be most effective in axons dorsal to NL (that is, from ipsilateral NM), which were not exposed to these chemorepulsive cues.

The expression of EphB receptors and ephrin-B ligands in both axon and target suggests several possible roles for EphB signaling. In addition to axon guidance, Eph-ephrin signaling, and in particular, EphB signaling, has a well-documented role in synaptogenesis and synaptic plasticity [48-61]. Because NM-NL synapses form at about E10, just after axons reach the neuropil area, the guidance of axons to these regions may be linked to formation of synapses in the correct regions. Moreover, forward and reverse signaling may influence each other due to interactions in *cis*, whereby ephrins can bind to Eph receptors within the same cell [62,63] or segregate laterally into distinct signaling domains [64,65]. These interactions can lead to downstream signaling and can alter the availability of either class for binding with proteins in *trans*[62,63,66,67].

### EphA and EphB signaling provide distinct axon guidance cues

In our fusion protein studies, we found that infusion of EphA4-Fc resulted in significantly more targeting errors in the NM-NL pathway than infusion of EphB1-Fc, suggesting that inhibition of EphA and EphB signaling is more effective than inhibition of EphB signaling alone. These results are consistent with the observation of extensive targeting errors with EphA4 electroporation [17] and suggest that in addition to EphB signaling and EphA4-ephrin-B2 interactions, EphA4 may facilitate axon guidance through interactions with ephrin-A ligands. We have previously demonstrated expression of ephrin-A2 in the auditory nerve and NL neuropil during the formation of NM-NL projections [45], indicating its feasibility as a candidate. Coordinated function between A and B classes has been shown to guide orderly projection patterns, notably in retinotectal projections [68-72]. Though we postulate here about loss of normal EphB2 repulsive cues, it is also likely EphA4 attractive cues are affected with EphA4-Fc injection and it would be interesting to explore this by analyzing ipsilateral NM-NL projections.

While our study focused on targeting of NM axons to distinct dorsal versus ventral NL regions, selective inhibition of the EphB class of receptors revealed an additional dimension of axon guidance for contralaterally projecting NM axons along the mediolateral axis. In particular, EphB fusion proteins resulted in pronounced lateral overgrowth by NM axons. Unlike the electroporation studies where misexpression was generally limited to NM axons and led to aberrant dorsoventral targeting, injection of fusion proteins into the hindbrain produced a broader inhibition that likely included EphB receptors in the NL neuropil. The observation of lateral overgrowth of NM axons using this approach may thus indicate that EphB signals arising in or near NL normally provide chemorepulsive cues that limit lateral growth.

### Morphogenesis of auditory nuclei

In addition to axon targeting errors, manipulations of Eph signaling also resulted in stereotyped morphological abnormalities. When EphB2 forward signaling was increased using plasmid electroporation at E2, prior to cellular migration and NL flattening into a monolayer, we observed a significant reduction in the size of NL. This effect may be a result of impaired migration of NL cells from the auditory anlage and/or from a reduction in total NL cells either by changes in cell fate specification or increased cell death. During normal development, NL undergoes extensive (84%) cell death as the monolayer forms [3]. Further analysis of changes in nuclei density and cell movement over time would be required to evaluate this possibility rigorously. NL was often disorganized, but in many cases the laminar appearance of NL was normal. Given that the majority of transfected cells were in NM, these results suggest a non-cell-autonomous role for EphB2 that would implicate NM-NL interactions in generating the appropriate morphology.

Our observations are consistent with previous reports that EphB/ephrin-B signaling has been shown to guide normal cellular migration in mammalian neocortex, hippocampus and cerebellum [73], avian and *Xenopus* neural crest [74-76], and zebrafish notochord [77] and hindbrain [78], whereby a contact repulsion mechanism is implicated [79,80]. EphB2 forward signaling in particular is responsible for lamination of hippocampal dentate gyrus cells, another brain region with distinct dorsal-ventral connectivity [81]. Though Eph-ephrin signaling is bidirectional [82], the abnormal morphologies were not seen with kiEphB2 transfection, where forward but not reverse signaling was impaired. Similarly disorganized NM-NL nuclei were also observed when EphA4 but not kiEphA4 was overexpressed [17] further implicating Eph receptor forward signaling in normal development. Together, these data suggest that forward signaling through EphA and EphB, possibly through their common ligand ephrin-B2, is necessary for the normal separation and organization of NM and NL. Indeed, inhibition of EphB forward signaling alone during E6 to E9 typically did not result in malformed nuclei, whereas inhibition of both subclasses together during E6 to E9 was sufficient to produce malformed nuclei at E10. Because NL lamination occurs when NM axons approach NL, it remains unclear whether either process is dependent on the other. Such an interaction is consistent with the observation that EphA4-Fc treated embryos often resulted in an abnormal NL morphology and also had a significant increase in targeting errors compared to EphB1-Fc treated embryos. Since multiple axons were often found crossing together following EphA4-Fc treatment, the possibility exists that fasciculation cues may also have been affected. However, because embryos with severely malformed nuclei did not meet inclusion criteria for axon targeting analysis, it is difficult to provide more than a qualitative correlation. Our findings suggest that integrated actions of Eph receptor signaling are necessary for migration of auditory nuclei precursor cells during development and that in turn, appropriate migration may be necessary for axonal target specification.

### Morphogenesis relies on Eph signaling at several developmental stages

When EphB forward signaling was inhibited using fusion proteins in *ex ovo* preparations from E6 to E9, though NL lamination appeared normal, we observed a tendency for NM axons to overshoot the lateral boundary of their contralateral NL target, suggesting that during normal development ephrin-B2 expressing axons may be limited to ventral NL neuropil by EphB2 forward signaling in NL cells. However, when forward signaling through both EphA and EphB was inhibited by EphA4-Fc injection, we observed more significant morphological defects. Similar to results for EphB2 electroporation, there was a reduced rostrocaudal extent of NL compared to controls. In contrast, the mediolateral extent was unchanged, suggesting that this axis is set earlier in development, while rostrocaudal extension may be more protracted.

The differences in morphological defects between treatment groups suggest that Eph proteins have distinct roles during different developmental phases. Effects seen with electroporation at E2 could result from early morphogenetic events, such as cell proliferation and formation of the auditory anlage from precursors in distinct regions [2,46], as well as later events, such as separation of NM and NL from the anlage and flattening of the NL cell body layer [15]. In contrast, effects of fusion protein infusion at E6 to E9 reflect only these later events. While electroporation targets mainly NM cells, fusion proteins diffuse broadly within the brainstem and may affect NM and NL as well as their surrounding regions. Thus the cell autonomy of these effects is difficult to determine. The observation that EphB2 receptor overexpression in NM led to morphologic defects in NL suggests a role for reverse signaling, but could also indicate changes in the levels of available ephrins in NM cells due to interactions with exogenous EphB2. Though the exact mechanisms involved here are unknown, perturbations to migratory pathways of neocortex, cerebellum and hippocampus are seen with loss of EphB2 and ephrin-B signaling and may be linked to changes in expression, recruitment and/or signaling of extracellular matrix proteins such as Reelin [73,81]. Migrating cortical neurons appear to use EphB versus EphA signaling differentially in determining radial versus tangential movement, respectively [80]. Likewise, our results suggest that Eph proteins have a significant role in the formation of the auditory brainstem circuit at several developmental time points, along discrete axes and for distinct developmental events.

## Conclusions

**Figure 7:**
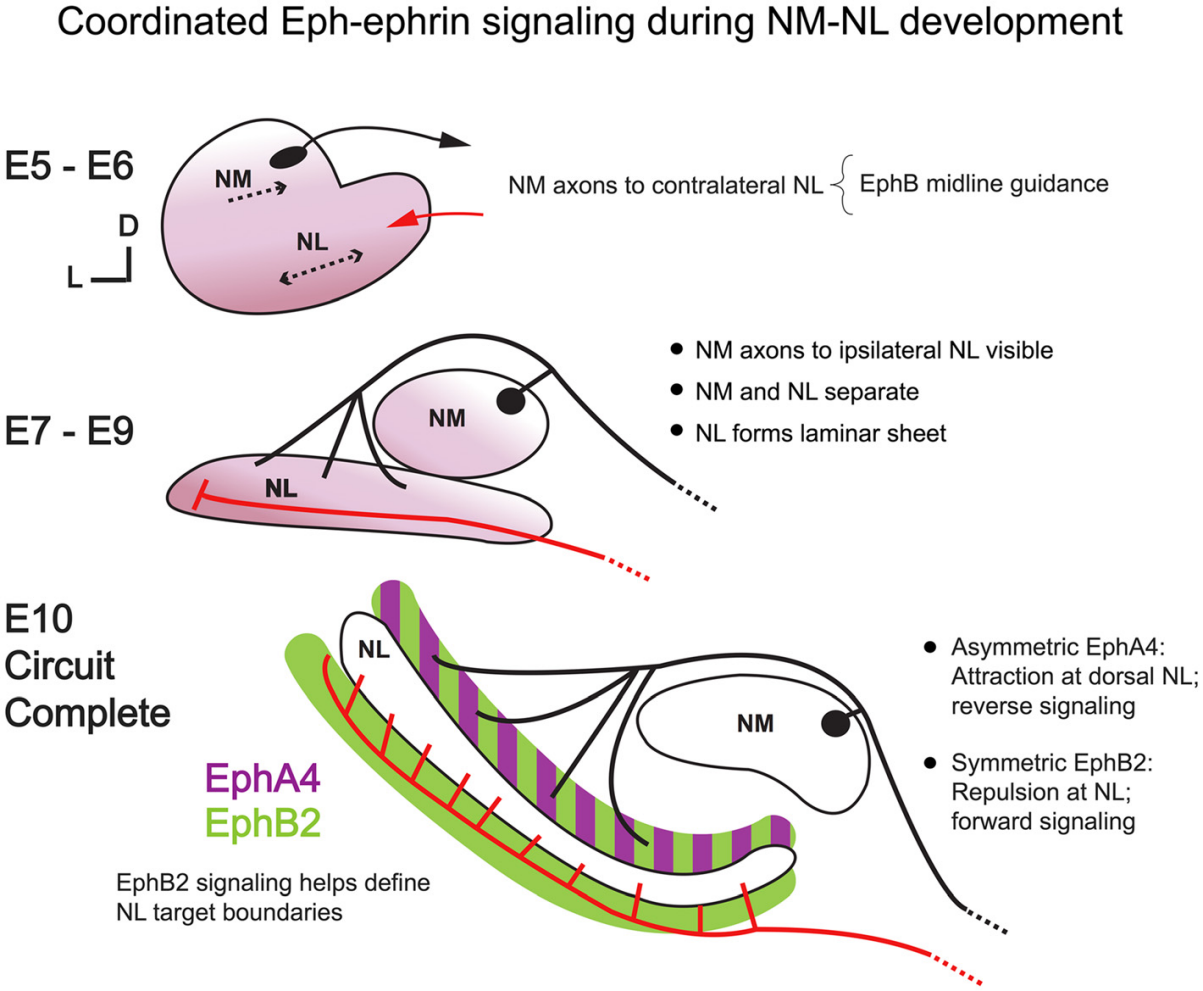
** Illustration of Eph-ephrin driven events important for normal development of the NM-NL circuit.** Left-sided NM and NL are schematized in a coronal view similar to sections shown in Figures1, 2, 3, 4, 5, 6 with dorsal up and medial to the right. Migratory and axon targeting events during embryonic days 5 through 10 (E5 to E10) are described. The black line represents ipsilateral NM projections (that is, those arising from the left side) and the red line represents contralateral NM projections (that is, those arising from the right side, not shown).

Our experimental results together with known developmental events seen during NM-NL development are summarized in Figure 7. The precursors for NM and NL coalesce in the auditory anlage by E5 [15,46]. In the protracted development of this pathway, NM and NL undergo morphogenetic changes while NM axons simultaneously grow toward their bilateral targets. By E10, precise topographic connections have formed with ipsilateral and contralateral contacts at distinct regions of respective dorsal and ventral dendrites. 

Together with previous expression and misexpression studies, our results highlight the complex interactions between Eph molecules in NM-NL development. In our model, we propose that EphA4 signaling in dorsal but not ventral NL neuropil is an attractive cue to ipsilateral NM axons, whereas the repulsive nature of EphB signaling acts at the NL cell body layer disallowing axons on either side from crossing the NL monolayer. The segregation of ipsilateral and contralateral NM inputs to NL could thus arise from the distinct expression patterns of EphA4 and EphB2 along with the opposing effects of these proteins on axon growth. This view is consistent with data on dorsoventral targeting (present study and [17]), midline axon guidance [13], and the data presented here on lateral growth of NM axons; it also takes into account the spatiotemporal expression of Eph family proteins in the developing pathway [27,44]. Several other factors must be considered before we can obtain a complete account of the mechanisms that give rise to this highly ordered neural pathway. Significantly, the link between morphogenesis of NM and NL and the concomitant growth of axons to precise targets is not understood. Our studies suggest that a small set of Eph proteins functions in both processes in distinct ways and that appropriate targeting could be dependent on maturation of the target. Though all Eph signaling inhibition treatments caused targeting errors, there were more errors when all signaling was blocked and unique morphologic outcomes associated with each condition. The increase in targeting errors when all Eph signaling was blocked versus EphB signaling alone might reflect an amplified effect of the same targeting mechanism, a result of the abnormal morphology *per se,* or both. While it is not yet possible to determine how these proteins act at each stage, or which other molecules contribute to the assembly of this circuit, our results, together with previous studies, suggest that coordinated Eph signaling at multiple steps is needed for the formation, connectivity and boundary specification of the NM-NL circuit.

## Methods

### Embryos

Fertilized brown Leghorn chicken eggs (*Gallus domesticus*) were obtained from AA Laboratories (Westminster, CA, USA) and stored at room temperature for up to two days prior to use. To initiate embryonic development, the eggs were placed in a rotating incubator at 39°C with a relative humidity above 70%. Chick embryos were either electroporated *in ovo* at E2 or transferred at E3 to a culture dish for *ex ovo* experimentation at E6 to E9. *Ex ovo* culture dishes were maintained in a non-rotating incubation chamber of similar temperature and humidity, allowing access to the developing embryo at later ages than the *in ovo* preparation. Each *ex ovo* culture preparation consisted of a disinfected polystyrene weigh dish (3.5 X 3.5 X 1 in.) into which the egg was opened, and a sterile square disposable petri dish bottom (100 X 100 X 15 mm) used as a lid. All embryos were sacrificed at E10 and the brainstem carefully dissected for *in vitro* whole mount axon tracing.

### *In ovo* electroporation

Eggs were removed from the rotating incubator at E2 and a small circular window was cut into the side of the eggshell following reinforcement with tape and removal of 3 mL thin albumin. Using a modified 25-guage hypodermic needle, a small amount of 4% India ink (in phosphate buffered saline (PBS)) was injected directly beneath the embryo to permit visualization. The vitelline membrane was removed with a fine tungsten needle and a small incision was made into the embryonic roof plate at the level of rhombomere 5 to provide access to the neural tube. Sterile PBS was placed over the area of interest to enhance electrical current and protect the embryo and underlying membranes from electrical burns. Paired needle-style gold-plated electrodes (Genetrodes, Harvard Apparatus, Holliston, MA USA) were positioned straddling the area of interest, approximately 2 to 3 mm apart. Plasmid DNA (1 to 3 μg/μL in Tris/EDTA), colored with a small amount of Fast Green dye, was injected into the neural tube opening using a 1.2 mm pulled glass pipette attached to a Picospritzer (Parker Hannifin Corporation, Irvine, CA USA). No more than 100 to 200 nL of plasmid DNA was delivered using a series of (typically 2 to 6) injections of 20 to 50 ms duration and 20 psi. Voltage was delivered using a BTX electroporator, with amplitude of 9 to 12 Volts and 50-ms duration, in trains of four to six pulses of 100 ms intervals. A total of 20 to 30 pulse trains were delivered for each embryo, with polarity switched between pulse trains in order to achieve bilateral transfection. All openings in the eggshell were sealed with tape, and the eggs were placed in a humid, non-rotating 39°C incubation chamber for an additional eight days.

### Plasmids

Plasmid vectors were used to introduce DNA via *in ovo* electroporation. Full-length EphB2 was cloned into pCAX at the EcoR1 site and co-transfected with pCAX-EGFP. The pCAX construct encodes chicken β-actin promoter, a CMV-IE enhancer, polycloning sites and poly-A signal. Full-length EphB2 or kinase inactive EphB2 (kiEphB2; provided by E. Pasquale, Sanford-Burnham Institute, La Jolla, CA, USA) containing a point mutation in the intracellular kinase domain was cloned into the pMES vector at the EcoR1 site. The pMES construct contains a chicken β-actin promoter, a CMV-IE enhancer and also encodes an EGFP reporter with an internal ribosomal entry site. As a negative control, embryos were transfected with pCAX-EGFP alone. Transfection was assessed by examination of EGFP fluorescence in dissected brainstems and in sectioned material using an epifluorescent microscope. In addition, we performed immunostaining on transfected embryos using methods described previously for EphA4 and EphB2 detection following electroporation [13,17,44].

### Recombinant fusion protein injections

A soluble recombinant protein containing the EphB1 receptor extracellular domain fused to the Fc region of Human IgG (rat EphB1-Fc; R&D Systems, Minneanapolis, MN, USA) was used to inhibit forward signaling through EphB receptors. Additionally, mouse EphA4-Fc (R&D systems) was used to inhibit forward signaling through EphA and EphB receptors. Recombinant proteins were diluted to 10 μg/mL in sterile 1X PBS and a small amount of methylene blue powder (Sigma-Aldrich, St. Louis, MO, USA) was dissolved into the solution at 37°C immediately prior to use to aid with injection visibility. Final solution was injected into the developing hindbrain at the floor of the fourth ventricle with a 1.2 mm pulled glass pipette attached to a Picospritzer using pulses of 20 to 200 ms duration and intensity of 20 psi. Injections were made for four consecutive days, E6 to E9, when NM axons have already crossed the midline and are approaching their contralateral NL target. The total volume injected varied from approximately 1.5 to 5.5 μL per embryo for respective E6 to E9 ages. Human IgG1-Fc (RD Systems) was used as a negative control. Fusion protein binding specificity was confirmed by incubation of fixed 30 μm cryosections with EphB1-Fc or IgG-Fc overnight, followed by secondary (anti-Fc) fluorescent antibody incubation for two hours.

### *Ex vivo *axon tracing

Axon tracing was used to identify and quantify targeting errors. E10 chick embryos were removed from the egg or the *ex ovo* culture dish and the brainstem quickly dissected in oxygenated artificial cerebrospinal fluid (aCSF; 130 mM NaCl, 3 mM KCl, 1.2 mM KH_2_PO_4_, 20 mM NaHCO_3_, 3 mM HEPES, 10 mM Glucose, 2 mM CaCl_2_, 1.3 mM MgSO_4_). A pulled glass micropipette with an approximately 10 μm opening at the tip, was filled with rhodamine dextran amine (RDA, MW = 3,000, Molecular Probes, Inc., Eugene, OR, USA) made at 6.25% with a 0.4% Triton X-100 in PBS, and attached by fine tubing to a Picospritzer. Using several pulses ranging from 10 to 50 ms at 20 psi, RDA was pressure injected into the dorsal midline of the medulla at the floor of the fourth ventricle to label only contralateral-projecting NM axons, as described previously [17]. The tissue was immersed in aCSF continuously infused with 95% O_2_/5% CO_2_ for 15 minutes. The tissue was then prepared for histologic sectioning as described in the next section and the axon tracings were later visualized by fluorescent microscopy.

### Histology

Tissue was fixed in 4% paraformaldehyde (PFA) in PBS for at least 2 hours, and then washed in 1X PBS for 10 minutes. For Vibratome sectioning, tissue was embedded in 2% low-melting agar (Fisher Scientific, Pittsburgh, PA, USA) and mounted to the stage, fully immersed in 1X PBS during sectioning. For cryostat sectioning, tissue was cryoprotected in 30% sucrose overnight prior to embedding in OCT medium. For all tissues, sequential 50 μm sections in the coronal plane were collected on a subbed glass slide and dried on a slide warmer at 37°C. Most sections were counterstained with the nuclear stain bisbenzimide (2 μg/mL in PBS, five minutes incubation followed by five minutes 1X PBS wash) to facilitate identification of NM and NL. Slides were coverslipped with Glycergel mounting medium (Dako, Carpinteria, CA, USA) and stored in the dark at 4°C until analyzed. A separate group of samples underwent immunofluorescence staining to confirm plasmid expression or fusion protein location. These samples were sectioned on the cryostat as described above into 25 μm sections.

### Immunofluorescence

Immunostaining was performed on a subset of samples to confirm plasmid expression with anti-EphB2 primary antibody or to confirm fusion protein localization with anti-Fc secondary antibody. Briefly, a hydrophobic pap-pen border was drawn around the sections and the slides were rinsed then incubated in blocking solution (4% goat serum, 0.01% Triton in 1X PBS) for one hour at room temperature in a humid chamber. The slides were quickly rinsed with PBS and then incubated with the primary antibody for confirmation of plasmid expression, or with secondary antibody for visualization of fusion protein localization. For slides undergoing primary antibody labeling, slides were rinsed after one day then incubated with the secondary antibody for labeling. All secondary antibody labeling was performed with Alexa Fluor anti-Fc antibodies (Molecular Probes, Inc., Eugene, OR, USA) used at a 1:1,000 dilution in blocking solution and incubated for two hours at room temperature in the dark.

### Image and data analysis

Experimental embryos with no gross abnormalities were used at E10 for data collection. Criteria for inclusion in further analysis were EGFP expression in the auditory brainstem of transfected embryos, four days of successful hindbrain injections for *ex ovo* cultured embryos, and successful midline axon tracing. Samples were coded and analyzed blind to experimental conditions. The slides were viewed on a Zeiss AxioSkop-2 epifluorescence microscope (Carl Zeiss, Thornwood, NY, USA) using 10X, 20X or 40X objective lenses. To quantify NM-NL axon targeting errors, NL was viewed at 20 and 40X across several focal planes in order to allow for systematic analysis of the entire width of every section. Targeting errors were defined as axons arising from contralateral NM that crossed the NL cell body layer to terminate in the dorsal neuropil, visible as a cell-free layer with bisbenzimide staining and dorsal to NL somata. At this age in development, NL was typically visible across at least eight sections, or 400 μm of coronally sectioned brain. Since the central region of this extent is where NL presents robustly as a single layer of cells (assuming no anatomical variation exists), this is the area where targeting errors can be most accurately quantified. Thus, for each embryo, n = 1 and a total of 400 μm of NL extent was analyzed, typically 200 μm (four adjacent sections X 50 μm each) from each left and right sides of the brain, at the central region along the NL axes. Mediolateral lengths were measured with Openlab software (Perkin Elmer, Waltham, MA, USA) by recording the distance between the medial-most and lateral-most borders of identifiable NL soma in equivalent sections. Rostrocaudal lengths were measured by counting the number of adjacent sections containing NL soma and multiplying by 50 (the thickness of each section). Left and right sides were recorded separately for anatomical measures. Once data collection was complete, the samples were decoded and grouped for comparison of means. Representative images were taken with Zeiss Axiocam digital camera and Openlab software. Any additional image analysis, tiling and color rendering or color merging was performed with Adobe Photoshop (Adobe, San Jose, CA, USA) and figures were prepared with Adobe Illustrator. All statistical analyses were done with JMP software (JMP, Cary, NC, USA) using a Student’s *t*-test and a significance value of *P* < 0.05.

## Abbreviations

ED: Embryonic day; ILDs: Interaural level differences; ITDs: Interaural time differences; NL: Nucleus laminaris; NM: Nucleus magnocellularis; PBS: Phosphate buffered saline; PFA: Paraformaldehyde.

## Competing interests

The authors declare that they have no competing interests.

## Authors’ contributions

MRA carried out the experiments and analysis for the electroporation and fusion protein studies. MRA and KSC contributed to the experimental design and manuscript preparation. Both authors have read and approved the final manuscript.
